# Cellular and Tissue Selectivity of AAV Serotypes for Gene Delivery to Chondrocytes and Cartilage

**DOI:** 10.7150/ijms.56760

**Published:** 2021-07-25

**Authors:** Dong Suk Yoon, Kyoung-Mi Lee, Sehee Cho, Eun Ae Ko, Jihyun Kim, Sujin Jung, Jae-Hyuck Shim, Guangping Gao, Kwang Hwan Park, Jin Woo Lee

**Affiliations:** 1 Department of Orthopedic Surgery, Yonsei University College of Medicine, Seoul 03722, South Korea; 2 Severance Biomedical Science Institute, Yonsei University College of Medicine, Seoul 03722, South Korea; 3 Brain Korea 21 PLUS Project for Medical Science, Yonsei University College of Medicine, Seoul 03722, South Korea; 4 Division of Rheumatology, University of Massachusetts Medical School, Worcester, MA 01605, USA; 5 Li Weibo Institute for Rare Diseases Research, University of Massachusetts Medical School, Worcester, MA 01605, USA; 6 Horae Gene Therapy Center, University of Massachusetts Medical School, Worcester, MA 01605, USA; 7 Department of Microbiology and Physiological Systems, University of Massachusetts Medical School, Worcester, MA 01605, USA; 8 Viral Vector Core, University of Massachusetts Medical School, Worcester, MA 01605, USA

**Keywords:** Adeno-associated virus (AAV) serotypes, Chondrocytes, Osteoarthritis, Gene therapy

## Abstract

**Background:** Despite several studies on the effect of adeno-associated virus (AAV)-based therapeutics on osteoarthritis (OA), information on the transduction efficiency and applicable profiles of different AAV serotypes to chondrocytes in hard cartilage tissue is still limited. Moreover, the recent discovery of additional AAV serotypes makes it necessary to screen for more suitable AAV serotypes for specific tissues. Here, we compared the transduction efficiencies of 14 conventional AAV serotypes in human chondrocytes, mouse OA models, and human cartilage explants obtained from OA patients.

**Methods:** To compare the transduction efficiency of individual AAV serotypes, green fluorescent protein (GFP) expression was detected by fluorescence microscopy or western blotting. Likewise, to compare the transduction efficiencies of individual AAV serotypes in cartilage tissues, GFP expression was determined using fluorescence microscopy or immunohistochemistry, and GFP-positive cells were counted.

**Results:** Only AAV2, 5, 6, and 6.2 exhibited substantial transduction efficiencies in both normal and OA chondrocytes. All AAV serotypes except AAV6 and rh43 could effectively transduce human bone marrow mesenchymal stem cells. In human and mouse OA cartilage tissues, AAV2, AAV5, AAV6.2, AAV8, and AAV rh39 showed excellent tissue specificity based on transduction efficiency. These results indicate the differences in transduction efficiencies of AAV serotypes between cellular and tissue models.

**Conclusions:** Our findings indicate that AAV2 and AAV6.2 may be the best choices for AAV-mediated gene delivery into intra-articular cartilage tissue. These AAV vectors hold the potential to be of use in clinical applications to prevent OA progression if appropriate therapeutic genes are inserted into the vector.

## Introduction

Gene delivery to cartilage tissue by intra-articular administration can serve as an attractive strategy for treating osteoarthritis (OA), except that vector access to hard cartilage tissue may be a limiting factor^1^. The ideal vector for introducing genes into hard cartilage tissue would enable sustained and stable gene expression in the tissue. Moreover, the vector could be injected using minimally invasive procedures without triggering serious vector-mediated inflammatory responses in the patients. In recent years, the use of adeno-associated virus (AAV) vectors has been considered the most promising strategy for delivering therapeutic genes.^2^ Not only can recombinant AAV be transduced into various tissues, but these vectors can also maintain AAV-induced gene expression for a long time without triggering an inflammatory response *in vivo*.^3^ To date, many studies have reported that AAV serotype capsids exhibit tissue specificities and different transduction efficiencies.^4^ As systemic and rational administration of AAV vectors derived from alternative serotypes is a powerful strategy for the successful delivery of therapeutic genes to target tissues, it is vital to determine an appropriate AAV serotype capsid that can be used for efficient transgene delivery. Several studies regarding the use of gene therapy for cartilage regeneration have shown promising results,^5^-^7^ but these studies used AAVs belonging to different serotypes. Potential differences between AAV serotypes may exist in experimental models of cells and tissues involving OA treatment or regeneration of damaged cartilage and should therefore be confirmed experimentally using a variety of *in vitro* and *in vivo* models, (human and mouse). It is also important to identify which cartilaginous cells and tissues are infected by AAVs after intra-articular injection and then use this information to develop ideal therapeutic strategies for intra-articular diseases such as OA.^5^

We aimed to determine the most efficient AAV serotype for the gene therapy of intra-articular diseases. We hypothesized that the transduction efficiency of individual AAVs in chondrocytes and cartilage tissues might differ in a serotype-dependent manner. To test this hypothesis, we used 14 conventional AAV vectors corresponding to different serotypes to identify the serotypes that can be efficiently delivered to chondrocytes within cartilage tissue. Our experiments revealed the differences in AAV serotype transduction efficiencies between chondrocytes and cartilage tissue. Our results revealed that AAV2 and AAV6.2 exhibited the highest transduction efficiencies, suggesting their use in AAV-mediated gene delivery into chondrocytes *in vitro* and *in vivo*. Furthermore, the corresponding vectors can be considered to represent optimal vector systems for clinical application in patients with intra-articular diseases, such as OA.

## Materials and Methods

### Cell culture

Human articular cartilage tissues were obtained from the knee joints of patients with approval from the institutional review board (IRB; 2019-1374-002) of the Yonsei University College of Medicine. Cartilage tissue was divided into undamaged and damaged parts, and chondrocytes were isolated from each tissue by incubating the tissues with 0.1% type II collagenase (Thermo Fisher Scientific, Rockford, IL, USA). Cells isolated from undamaged cartilage tissues were designated as normal chondrocytes, and the cells isolated from damaged cartilage tissues were considered as osteoarthritic (OA) chondrocytes. Chondrocytes were cultured in high-glucose DMEM (DMEM-HG; Invitrogen, Carlsbad, CA, USA) supplemented with 10% fetal bovine serum (FBS; Gibco, Grand Island NY, USA) and 1% antibiotic-antimycotic solution (Invitrogen) at 37°C and 5% CO_2_. To obtain bone marrow mesenchymal stem cells, bone marrow aspirates were obtained from the posterior iliac crests of 12 adult donors, with the approval from the IRB (2017-0308-001) of the Yonsei University College of Medicine. Cell culture was performed, as described previously.^8^ ATDC5 mouse chondrocyte cells were purchased from Sigma-Aldrich (St. Louis, MO, USA). ATDC5 cells were maintained in high-glucose DMEM (DMEM-HG; Invitrogen) supplemented with 10% FBS and 1% antibiotic-antimycotic solution at 37°C and 5% CO_2_.

### AAV vector preparation and *in vitro* transduction

All AAV serotypes (AAV1-9, 6.2, rh8, rh10, rh39, and rh43) were produced from the Viral Vector Core at University of Massachusetts Medical School. AAV vectors use self-complementing and the chicken beta-actin promoter. AAV production in this study involved transient transfection of HEK293 cells, purification by CsCl sedimentation, and titration by droplet digital PCR (ddPCR) on a QX200 ddPCR system (Bio-Rad) using the *Egfp* prime/probe set, as previously described.^9^, ^10^ Human normal or OA chondrocytes, ATDC5 cells, and mesenchymal stem cells (MSCs) were seeded at a density of 1 × 10^4^ cells per well in 24-well plates, and 24 h later, the cells were incubated with AAV1, 2, 3, 4, 5, 6, 7, 8, 9, 6.2, rh8, rh10, rh39, or rh43 vectors packaging the green fluorescence protein (GFP) reporter transgene at 6000 MOI (1 X 10^9^/mL genome copies [GC]). After 48 h, GFP fluorescence was monitored using a fluorescence microscope to determine the efficiency of AAV transduction. Dose-response experiments were not conducted in the current study because the appropriate MOI for the AAVs was already established in a previous study.^9^

### Western blotting

Cells transduced with individual AAV serotypes were lysed using PRO-PREP^TM^ Protein Extraction Solution (iNtRON Biotechnology, Seongnam, South Korea). Protein concentrations were determined using the Bio-Rad Protein Assay (Bio-Rad Laboratories, Inc., Hercules, CA, USA). Protein samples (30 μg) were resolved by SDS-polyacrylamide gel electrophoresis (SDS-PAGE; Sigma-Aldrich) on a 10% gel. The resolved proteins were transferred onto membranes and blocked with 5% skim milk (BD, Sparks, MD, USA) for 1 h at room temperature. The membranes were probed for ~12 h using anti-GFP antibodies (Santa Cruz Biotechnology, Santa Cruz, CA, USA). Membranes were further probed with antibodies against HSP90 (Santa Cruz Biotechnology), which served as a loading control.

### Animal experiments and *in vivo* transduction of AAV

All animal experiments were approved by the Institutional Animal Care and Use Committee (IACUC) protocol of the Yonsei University College of Medicine (approval number: IACUC-2016-0099). 1 x 10^10^ GC (10 μL) of each AAV serotype was injected intra-articularly to confirm the transduction efficiencies in the cartilage tissue of mice. Mice were fed an alfalfa-free chow diet to minimize GFP fluorescence following analyses. Two weeks later, the mice were euthanized, and the joints were collected for histological assessment (n = at least 6 per group). To confirm whether individual AAVs were transduced into intra-articular tissues, the collected samples were visualized using an animal optical imaging system (IVIS, Caliper Life Sciences, MA, USA). Scanning was performed under anesthesia at wavelengths of 744 and 805 nm. The fluorescence intensity in the histological samples from each mouse was calculated from the fluorescence signal (p/s/cm2) using Living Image software version 2.50 (Xenogen). To ensure reproducibility, all tests were performed in at least three mice.

### Histology and immunohistochemistry

Collected mouse joint tissues were fixed for 7 days in 10% formalin at room temperature. For human samples, cartilage explants were obtained from the articular cartilage of patients (three females; aged 60~70) undergoing total knee arthroplasty. Sample collection was performed with patient consent after obtaining approval from the IRB (2019-1374-002) of the Yonsei University College of Medicine. The cartilage tissues were immersed in PBS at the surgical operating room and transferred immediately to DMEM/high glucose medium containing 10% FBS. Cartilage explants were punched into full-thickness cylindrical explants with a diameter of 5 mm and a length of 1 mm using a stainless-steel punch (Miltex Instruments, PA, USA). The prepared cartilage explants were incubated in DMEM/high glucose medium containing 10% FBS and treated immediately with each AAV at a concentration of 1 X 109 GC/mL. The cultures were maintained for 72 hours and then fixed with 10% formalin at room temperature. After fixation, the formalin-fixed specimens were embedded in paraffin. The paraffin-embedded sections were deparaffinized, rehydrated, and washed with PBS, and the tissue sections were used to evaluate the transduction efficiencies of individual AAV serotypes in mouse cartilage tissues. The prepared tissue samples were sliced to a thickness of 4 μm and probed with the anti-GFP (Santa Cruz Biotechnology) antibody to detect GFP levels. To visualize GFP expression in the anti-GFP-antibody‒probed tissues, the samples were stained with aminoethyl carbazole (AEC; Abcam, Cambridge, UK). The stained tissues were observed using a VS120 virtual microscope (Olympus, Tokyo, Japan), and images of the sections were analyzed using the OlyVIA 2.5 program (Olympus).

### Statistical analyses

All experiments were performed in triplicate using samples from at least three donors. Data are depicted as the mean ± standard deviation.

## Results

### AAV serotypes show different transduction efficiencies in chondrocytes and mesenchymal stem cells

**Figure 1:**
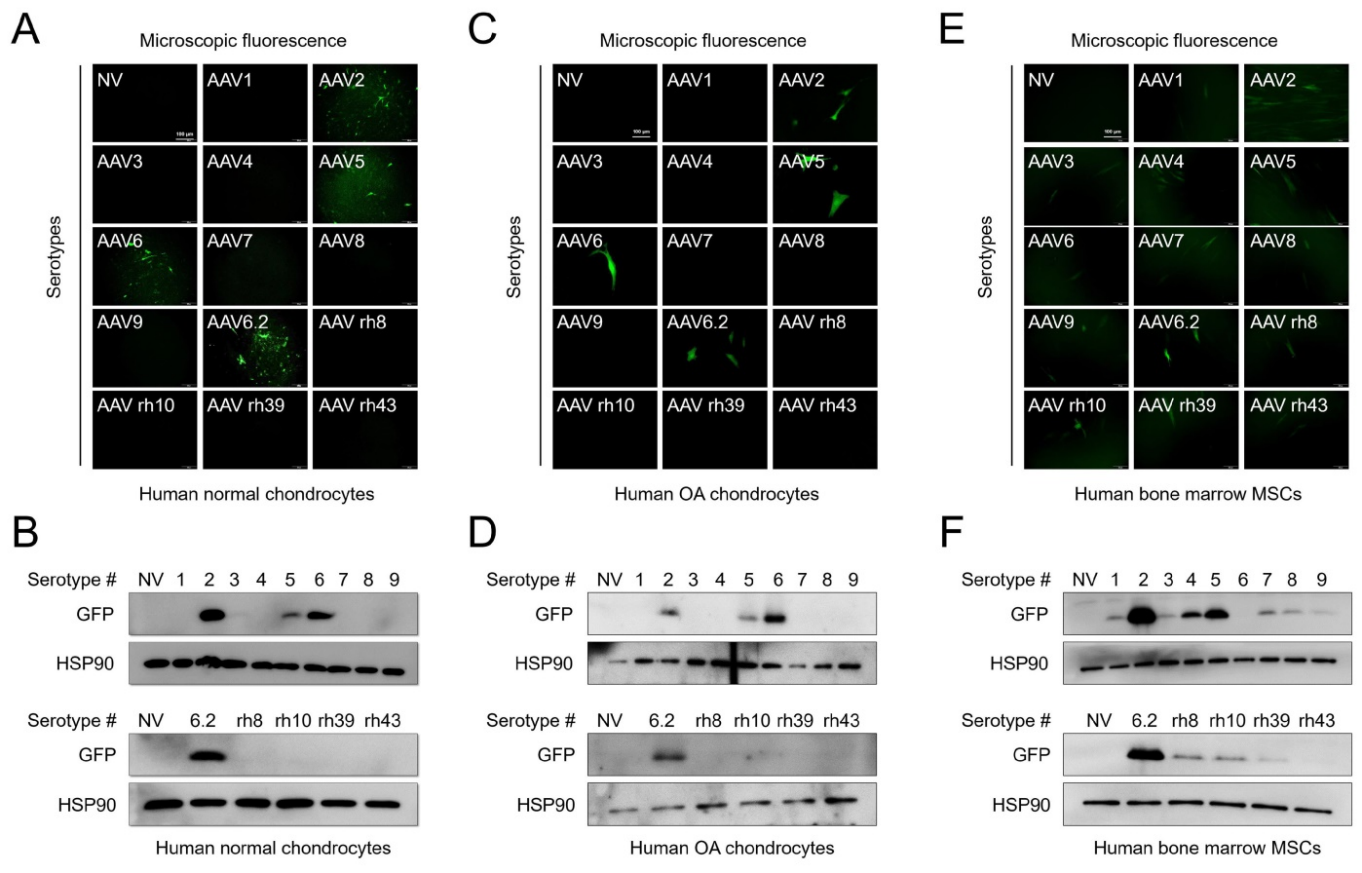
** Transduction efficiency of individual AAV serotypes within different cell culture models:** (**A**) Representative GFP fluorescence images in human normal chondrocytes 2 days after transduction of individual AAV vectors. (Scale bar = 100 µm). (**B**) Western blot analysis of GFP expression 48 h after AAV transduction into normal human chondrocytes. (**C**) Representative GFP fluorescence images in human OA chondrocytes 2 days after transduction with individual AAV vectors. (Scale bar = 100 µm). (**D**) Western blot analysis of GFP expression 48 h after AAV transduction into human OA chondrocytes. (**E**) Representative GFP fluorescence images in human bone marrow MSCs 2 days after AAV transduction. (Scale bar = 100 µm). (**F**) Western blot analysis of GFP expression 48 h after AAV transduction into human bone marrow MSCs. NV, no vector; AAV, adeno-associated virus; rh, rhesus; OA, osteoarthritis; GFP, green fluorescence protein; HSP, heat shock protein 90; MSC, mesenchymal stem cells.

We first compared the transduction efficiency of AAV serotypes with different capsid sequences. Normal human chondrocytes were obtained from an intact part of cartilage explants from patients who had undergone surgery, and human OA chondrocytes were obtained from damaged regions of cartilage explants from patients who had undergone surgery. The cells were transduced with individual AAV serotypes and observed under a fluorescence microscope after 48 h. To further determine the transduction efficiency of individual serotypes, the expression of green fluorescence protein (GFP) (AAV vectors were GFP tagged) was determined by western blotting. GFP expression was detected in both normal and OA chondrocytes transduced with AAV2, 5, 6, and 6.2 serotypes (Figure 1A, C). Likewise, GFP expression was only detected in AAV2-, 5-, 6-, and 6.2 serotype-transduced cells (Figure 1B, D). However, in human bone marrow-derived MSCs, most of the AAV serotypes were transduced except for AAV6 and rh43 (Figure 1E, F). These results indicate that AAV serotypes are cell-type‒specific. Therefore, we propose that AAV2, 5, 6, and 6.2 are suitable serotypes for human chondrocyte-based *in vitro* culture systems. Additionally, we tested the transduction efficiency of AAV serotypes in the mouse chondrocyte cell line, ATDC5. We found that GFP fluorescence and expression were strongly detected in AAV5- and 6.2-transduced ATDC5 cells, whereas the fluorescence and expression of GFP were weakly detected in AAV2-, 6-, 7-, rh10-, rh39-, and rh43-transduced cells (Figure S1A, B), indicating that AAV serotypes may also be species-type‒specific.

### Tissue-specific transduction by each AAV serotype

**Figure 2:**
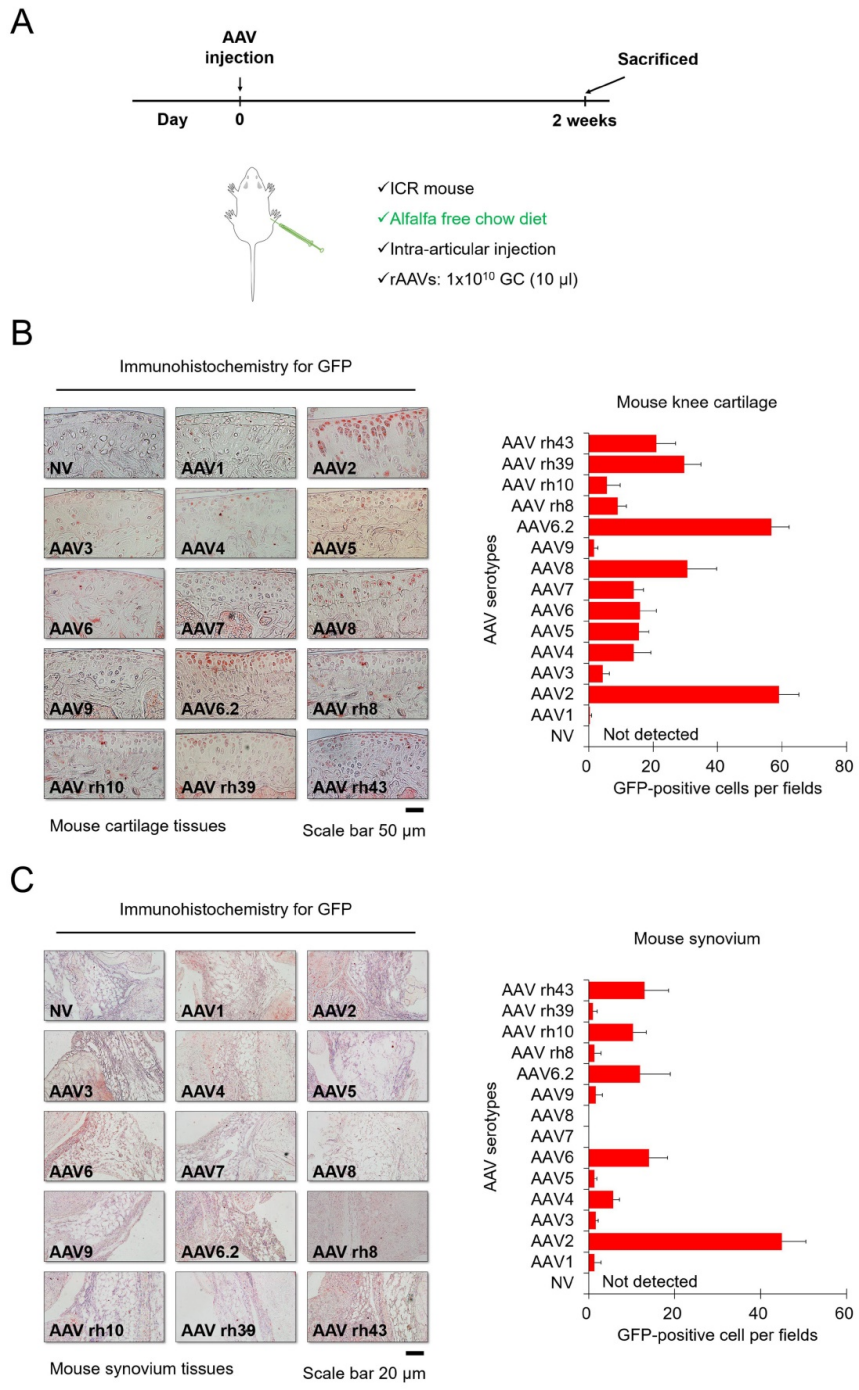
** Transduction efficiency of individual AAV serotypes in mouse cartilage tissue:** (**A**) Schematic of animal experiments performed in this study. Mice were fed an alfalfa-free chow diet to minimize the background fluorescence. Individual AAV serotypes were injected at a dose of 1 x 10 GC/10 μL. (**B**) Representative images of GFP (red) immunostaining in joint cartilage sections obtained from the knees of mice injected with individual AAV serotypes. (Scale bar = 50 µm). Quantification of GFP-positive cells in individual AAV serotype-infected joint sections. (**C**) Representative images of GFP (red) immunostaining in synovial membrane sections obtained from the knees of mice injected with individual AAV serotypes (Scale bar = 20 µm). Quantification of GFP-positive cells in individual AAV serotype-infected synovium sections. Data are expressed as a mean ± SD. GC, genome copies.

**Figure 3:**
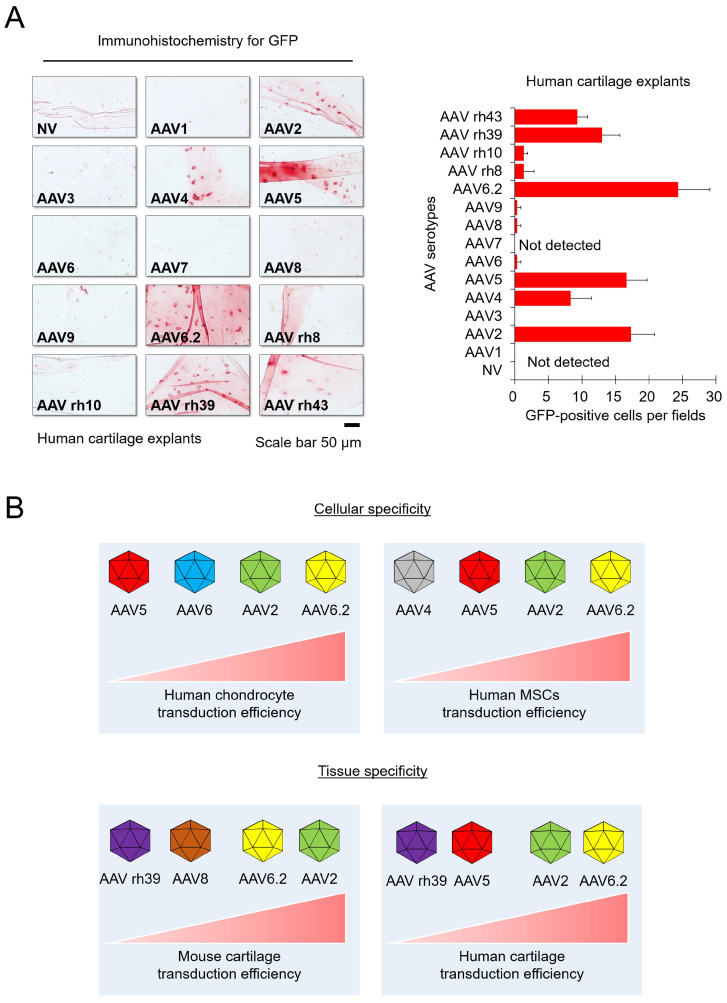
** Transduction efficiency of individual AAV serotypes in human cartilage explants:** (**A**) Representative images showing GFP expression (red) in human cartilage explants transduced with individual AAV serotypes. (Scale bar = 50 µm). Quantification of GFP-positive cells in individual AAV serotype-infected cartilage explant sections. Data are expressed as the mean ± SD. (**B**) Schematic of the cellular and tissue specificity of individual AAV serotypes tested in this study. In this study, AAV5 and AAV6.2 are effective in both cell and animal models.

Next, we injected each AVV serotype into the knee joints of mice to determine the corresponding transduction efficiencies, and mice were fed alfalfa-free chow to minimize background fluorescence. Each AAV serotype was injected intra-articularly, and then the mice were euthanized for further experiments after 2 weeks (Figure 2A). Figure 2B shows immunohistochemistry data for GFP expression within cartilage tissues. In the group injected with most AAV serotypes except for AAV1 and AAV9, GFP was detected in the superficial zone of cartilage tissues of the mouse knee (Figure 2B). In particular, AAV2 and AAV6.2 showed greater transduction efficacy on chondrocytes residing within hard cartilage tissues, compared to other AAV serotypes. Our data from *in vitro* cell culture models also showed that AAV2 and AAV6.2 had great transduction efficiency in human chondrocytes and MSCs, suggesting that AAV2 and AAV6.2 are more specific for chondrocytes under both *in vitro* and *in vivo* environments. Altogether, these results demonstrated that AAV2 and AAV6.2 may be the best candidate vectors that can efficiently deliver therapeutic genes to chondrocytes. The introduction of therapeutic genes into the synovial membrane of the joint could a strategy for the treatment of OA^11^ Figure 3C shows immunohistochemistry results for GFP expression within the synovia in mouse knee joints. While AAV6, AAV6.2, AAV rh10, and AAV rh43 showed some transduction efficacy, AAV2 exhibited greater transduction efficiency than the others in the synovial membrane of the mouse knee joints (Figure 2C).

To extrapolate our experimental results to humans, human cartilage explants were obtained from patients undergoing surgical procedures. AAV transduction experiment with human articular cartilage tissue was done under the *in vitro* environment with explants. The human cartilage explants were cultured in chondrocyte culture medium containing individual AAV serotypes for 3 days, and then frozen sections were used for immunohistochemistry. We confirmed that GFP expression was detected in the AAV2-, AAV4-, AAV5-, AAV6.2-, AAV rh39-, and AAV rh43-treated groups. In particular, the AAV2- and AAV6.2-treated groups display strong GFP expression, similar to the results in mice experiments (Figure 3A). These results indicated that AAV2 and AAV6.2 may be suitable for *in vitro* and *in vivo* studies, as well as for applications in human OA patients.

## Discussion

Gene therapy is a promising treatment option for musculoskeletal diseases.^1^, ^12^, ^13^ Due to their versatility, non-pathogenic properties, high gene delivery efficiency, and long-term persistence, AAVs are anticipated to be used extensively in the treatment of musculoskeletal diseases,^14^, ^15^ indicating their high clinical value. Several studies have confirmed that AAVs can deliver genes to the articular joint of mice, and some studies have attempted to compare different serotypes of AAV to evaluate their transduction efficiencies in articular tissues*.*^16^-^18^ As articular cartilage has a specialized architecture and limited regeneration capacity, the delivery of genes into articular chondrocytes is challenging. One way of overcoming this difficulty is to choose the most efficient vector for therapeutic gene delivery. In this study, we showed that AAV2 and AAV6.2 could effectively deliver therapeutic genes into human joint tissues. Others have reported that AAV2 has the highest transduction efficiency in the cartilage of knee joints 19, it has been used in clinical trials in patients with inflammatory arthritis.^20^ Collectively, these studies support the current data that AAV2 is one of the most efficient vector systems with which to transfer therapeutic genes into chondrocytes of human joint cartilage. Our results also showed that AAV6.2, an AAV6F129L point mutant, may be very effective in transducing therapeutic genes into human joint tissue (Figure 3). AAV6.2 was found to be 2-fold more efficient than AAV6 in transducing mouse airway epithelium.^21^ Another study showed that a triple AAV6 mutant, termed AAV6.2FF, was 10-fold more efficient than AAV6 for transgene expression in the lung.^22^ Our results also showed that AAV6.2 was approximately four times more efficient than AAV6 in the transduction of human cartilage explants when the experiments were conducted under the *in vitro* conditions (Figure 3). This is the first study to report the high transduction efficiency of AAV6.2 for transgene delivery into human chondrocytes as well as cartilage tissue. Therefore, we propose that AAV2 and AAV6.2 are viable options for OA-related studies as well as future clinical approaches for human application because of their cellular and tissue specificity.

Despite the many ideal results of AAV delivery systems into joint tissues, there are several considerations associated with the clinical setting. Different tissues, such as the synovium, in the joint cavity could be exposed to AAV injection. To solve this problem, one alternative is to incorporate a peptide into the AAV vector to achieve tissue specificity. Indeed, many researchers have tried to insert tissue-specific peptides to increase the transduction efficiency and tissue specificity for successful AAV-based therapies.^9^, ^23^ We are also trying to develop the AAV2 and AAV6.2 vectors with chondrocyte-binding peptides to enhance the tissue specificity and improve the efficiency of AAV-based gene therapy. These attempts will help in applying AAV-based gene therapy for OA in the future. The current study is meaningful in that AAVs with high transduction efficiencies for transgene delivery in joint tissue were compared and determined among various AAV serotypes.

## Conclusion

In conclusion, the current study suggests that the use of AAV2 and AAV6.2 vectors may confer the best transduction efficacy in human articular cartilage tissue, compared to other commonly used AAV vector serotypes. The information provided herein support the selection of clinically suitable AAV serotypes in clinical trials of OA patients. Overall, we propose that intra-articular delivery of AAV2 or AAV6.2 vectors inserted with an appropriate therapeutic gene may be a promising therapeutic approach for preventing OA progression.

## Supplementary Material

Supplementary figure.
