# Supplementary material for: Cellular and Tissue Selectivity of AAV Serotypes for Gene Delivery to Chondrocytes and Cartilage
Source: Int J Med Sci. 2021 Jul 25;18(15):3353–60. doi: 10.7150/ijms.56760 (PMC8436087; doi:10.7150/ijms.56760)
Supplement: Supplementary file 1 — Supplementary figure. [file ijmsv18p3353s1.pdf]

SUPPLEMENTARY DATA

Cellular and tissue selectivity of AAV serotypes for gene delivery to chondrocytes and cartilage

Dong Suk Yoon[1#], Kyoung-Mi Lee[1,2#], Sehee Cho[1,3#], Eun Ae Ko[1], Jihyun Kim[1], Sujin Jung[1], Jae-Hyuck Shim[4,5,6], Guangping Gao[5,6,7,8], Kwang Hwan Park[1], Jin Woo Lee[1,2,3]

[1]Department of Orthopedic Surgery, Yonsei University College of Medicine, Seoul 03722, South Korea

[2]Severance Biomedical Science Institute, Yonsei University College of Medicine, Seoul 03722, South Korea

[3]Brain Korea 21 PLUS Project for Medical Science, Yonsei University College of Medicine, Seoul 03722, South Korea

[4]Division of Rheumatology, University of Massachusetts Medical School, Worcester, MA 01605, USA

[5]Li Weibo Institute for Rare Diseases Research, University of Massachusetts Medical School, Worcester, MA 01605, USA

[6]Horae Gene Therapy Center, University of Massachusetts Medical School, Worcester, MA 01605, USA

[7]Department of Microbiology and Physiological Systems, University of Massachusetts Medical School, Worcester, MA 01605, USA

[8]Viral Vector Core, University of Massachusetts Medical School, Worcester, MA 01605, USA

# DSY, K-ML, and SC contributed equally to this work and are co-first authors to this article.

Corresponding authors: Professor Jin Woo Lee, Department of Orthopaedic Surgery, Yonsei University College of Medicine, Seoul 03722, South Korea; ljwos@yuhs.ac (Tel: +82 2 2228 2190) and Professor Kwang Hwan Park, Department of Orthopaedic Surgery, Yonsei University College of Medicine, Seoul 03722, South Korea; khpark@yuhs.ac (Tel: +82 2 2228 2185)

SUPPLEMENTARY FIGURES

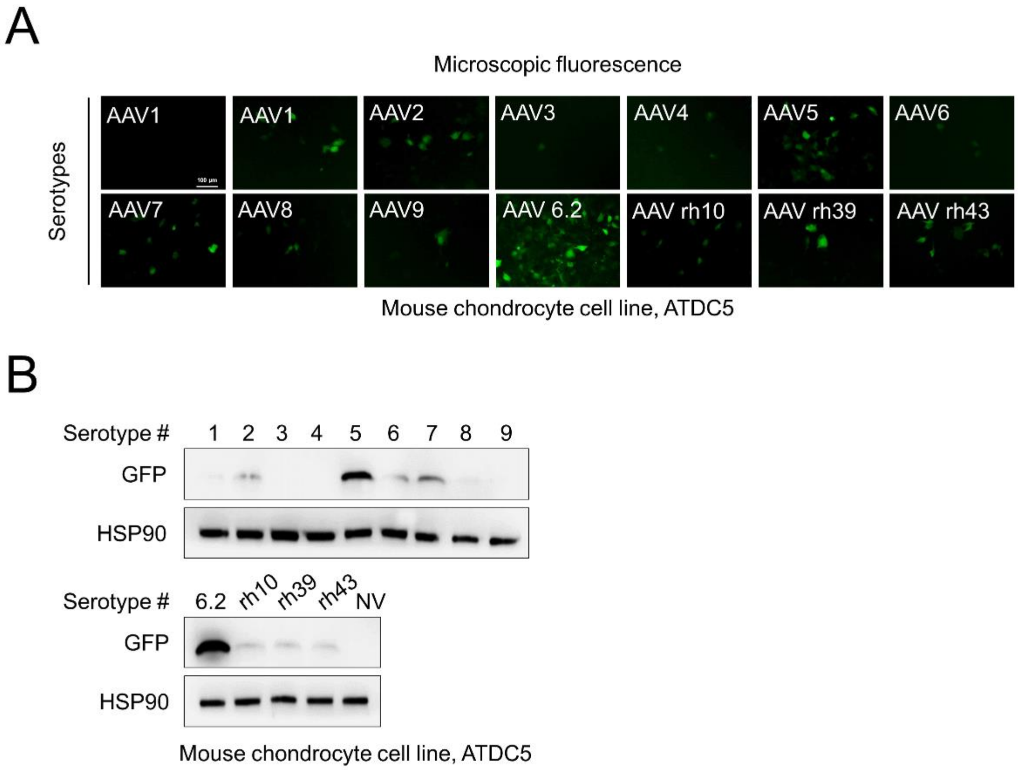

Figure S1. Transduction efficiency of individual AAV serotypes in ATDC5 cells. (**A**) Representative fluorescence images depicting GFP expression in ATDC5 cells 2 days after transduction with individual AAV serotypes. (Scale bar = 100 µm). (**B**) Western blot analysis of GFP expression 48 h after ATDC5 cells were transduced with individual AAV serotypes.
